# Sphingosine kinase 2 inhibitor ABC294640 suppresses neuronal excitability and inhibits multiple endogenously and exogenously expressed voltage-gated ion channels in cultured cells

**DOI:** 10.1080/19336950.2020.1788364

**Published:** 2020-07-13

**Authors:** Fei Zhang, Wenqi Hu, Lili Qu, Chunlei Cang

**Affiliations:** Division of Molecular Medicine, Hefei National Laboratory for Physical Sciences at Microscale, CAS Key Laboratory of Innate Immunity and Chronic Disease, Neurodegenerative Disorder Research Center, School of Life Sciences, University of Science and Technology of China, Hefei, Anhui, China

**Keywords:** Sphingosine kinase 2, ABC294640, potassium channel, sodium channel, calcium channel, action potential

## Abstract

Sphingolipids regulate multiple cellular processes, including proliferation, autophagy, and apoptosis. Sphingosine kinases, the key enzymes in the metabolism of sphingolipids, are overexpressed in many cancers, making them important targets for the development of antitumor drugs. ABC294640 is a selective sphingosine kinase 2 (SK2) inhibitor that shows good antitumor activity in vitro. One phase I clinical study of ABC294640 reported that ABC294640 caused a variety of neurological disorders. The mechanism of these phenomena, however, remains unclear. In the present study, we used in vitro cell experiments to test the effects of ABC294640 on the nervous system. We found that ABC294640 suppressed the firing of action potentials in cultured hippocampal neurons from neonatal mice and inhibited endogenous sodium, potassium, and calcium currents in both cultured neurons and SH-SY5Y cells. In addition, we tested four types of human voltage-gated potassium channels transiently expressed in HEK293T cells. All were inhibited by ABC294640, of which K_V_4.2 and K_V_1.4 were more sensitive than BK and K2P2.1. The effect of ABC294640 on ion channels was different from another SK2 inhibitor K145 and was not affected by S1P. The fast onset and recovery of the inhibition indicated that ABC294640 was likely to inhibit ion channels by acting directly on channel proteins, rather than by inhibiting SK2. These results revealed the mechanism by which ABC294640 interferes with the nervous system. To develop future antitumor drugs, researchers should modify the structure of ABC294640 to avoid its effects on ion channels or should develop compounds that target SK2 or downstream molecules.

## Introduction

Sphingosine and ceramide are crucial mediators of sphingolipid metabolism and biosynthesis. Sphingosine can be generated through ceramide de-acylation and subsequently can be phosphorylated by sphingosine kinase, which has two isoforms (SK1 and SK2), to produce sphingosine-1-phosphate (S1P). In turn, S1P regulates cell growth, migration, survival, proliferation, and immune responses [1,2]. Many studies have indicated that S1P promotes the motility, survival, growth, and transformation of cancer cells through multiple pathways [3,4]. In addition, a variety of human cancers involve ectopically expressed SK1 and SK2. As such, sphingosine kinase is an important clinical target for tumor treatment [5–8].

As a selective inhibitor of SK2, ABC294640 has displayed antitumor activity in many cancer models [9]. For example, in a lymphoblastic leukemia cell model, ABC294640 inhibited expression of both MYC and c-Myc–regulated genes; it also reduced cell proliferation and survival [10]. In non-small-cell lung cancer, it enhanced the antitumor effects of TRAIL by inducing apoptosis [11]. Furthermore, in the treatment of chemo-resistant and endocrine therapy–resistant breast cancer, ABC294640 may be a viable antitumor therapeutic [12].

In one phase I clinical trial, patients with advanced solid tumors experienced neurological disorders, including dizziness, paresthesia, memory loss, anxiety, hallucinations, and other symptoms of nerve function disturbance after ABC294640 treatment. Some symptoms resolved after interruption or discontinuation of ABC294640 [13]. This suggested that ABC294640 has a regulatory effect on the nervous system, but the molecular mechanisms of that effect remain unclear.

In this study, we found that ABC294640 suppressed the firing of neuronal action potential (AP) and inhibited multiple ion channels. These results revealed the molecular mechanisms of ABC294640-induced neurological disorders and provided insight for the future development of antitumor drugs.

## Materials and methods

### Cell culture

Cells were maintained at 37°C in a humidified CO_2_ incubator. SH-SY5Y cells and HEK293T cells were cultured in Dulbecco’s modified Eagle’s medium (DMEM) (Gibco) supplemented with 10% fetal bovine serum (FBS) (Biological Industries), 1× glutagro supplement (Corning), and 1**×** penicillin/streptomycin (Biosharp). To record calcium currents, 10 μM all-trans-retinoic acid was added to the SH-SY5Y culture for at least 24 h to induce differentiation. Cells used for patch clamp recording were plated on coverslips 12 h before the experiment and transferred to a perfusion chamber before recording. Cells used for calcium imaging were seeded on 35 mm culture dishes with thin-glass bottoms. Hippocampal neurons were cultured from P0 neonatal C57BL/6N mice. The use of mice complied with the National Institutes of Health guide for the care and use of laboratory animals and was approved by the Animal Care and Use Committee of the University of Science and Technology of China. Briefly, the mouse hippocampi were isolated from the brain and cut into small pieces. The tissues were then digested using 20 units/mL of papain (Worthington) at 37°C for 30 min. Cells were then dissociated by pipetting up and down and cultured in DMEM/F12 supplemented with 10% FBS (Biological Industries), 0.5**× **penicillin/streptomycin (Biosharp), and 1**× **GlutaMAX (Gibco). After 24 h, the culture medium was changed to Neurobasal-A (Gibco) supplemented with 1**× **B-27 (Gibco), 0.5**× **penicillin/streptomycin (Biosharp), and 1**×** GlutaMAX (Gibco). The neurons were used for experiments at DIV 8–15.

### cDNA constructs and transfection

cDNA constructs of human *KCNA4* and *KCND2* were purchased from Sino Biological and Miaolingbio, respectively. The cDNA sequence of *KCNA4* was subcloned into the pCMV3 vector. The cDNA sequence of *KCND2* with FLAG tag was subcloned into the PLVX-IRES-ZsGreen1 vector. The cDNA constructs of mouse *KCNMA1* and *KCNK2* were purchased from Zoman Biotechnology. The cDNA sequences of *KCNMA1* and *KCNK2* were subcloned into the pECMV vector. All constructs were transfected into HEK293T cells using jetPRIME (Polyplus-transfection) reagent according to the manufacturer’s instructions.

### Compounds

ABC294640 (MedChemExpress) was first dissolved in dimethyl sulfoxide (DMSO) to make a 30 mM stock solution. The working concentration was achieved by adding appropriate amount of stock solution to the bath solution. Vortex was used to facilitate dissolving of ABC294640. Same amount of DMSO was included in the bath solution for the control experiments. In addition, we also tested the effect of DMSO on the channel currents. The highest dose of DMSO (0.2%, v/v) used in our patch-clamp experiments had no effect on the peak and persistent K_V_1.4 currents (data not shown). K145 (MedChemExpress) was dissolved in H_2_O to make a 10 mM stock solution. S1P (Cayman Chemical) was dissolved in 0.3 M NaOH solution to make a 10 mM stock solution.

### Electrophysiology

Whole-cell patch-clamp recordings were performed using an Axopatch 200B patch-clamp amplifier, a Digidata 1550B data acquisition system, and pClamp software (Molecular Devices). Recording pipettes were made from borosilicate glass tubes using a P-1000 puller (Sutter Instrument). Unless otherwise indicated, the bath solution used in the voltage-clamp experiments contained 135 mM NaCl, 5.4 mM KCl, 10 mM HEPES, 1.8 mM CaCl_2_, 0.9 mM MgCl_2_, and 10 mM D-glucose (pH 7.40 by NaOH), and the pipette solution contained 130 mM K-aspartate, 25 mM KCl, 1 mM EGTA, 5.5 mM Mg-ATP, and 5 mM HEPES (pH 7.20 by KOH). To record sodium currents, 10 mM tetraethylammonium (TEA) was added to the bath to inhibit endogenous potassium channels. To record BK channels (Figure 6(a–e)), 1.33 mM CaCl_2_ was added to the pipette solution to activate the channel. To record calcium currents (Figure 4(a–b) and Figure 7(d–e)), the bath solution contained 115 mM choline chloride, 10 mM BaCl_2_, 2 mM MgCl_2_, 10 mM HEPES, 10 mM glucose, 5 mM TEA, and 0.003 mM tetrodotoxin (pH 7.4 by CsOH), and the pipette solution contained 120 mM CsCl, 20 mM TEA, 10 mM EGTA, 2 mM MgCl_2_, 10 mM HEPES, and 2 mM ATP-Mg (pH 7.2 by CsOH). The perfusion of bath solution or bath solution containing ABC294640 were done using a VC-8P perfusion system and an RC-26 recording chamber (Warner Instruments).

The voltage dependence of activation (Figure 5(d,k)) was studied using a 200 ms voltage-step protocol (−80 to +20 mV, 10 mV step; Vh = −100 mV for K_V_4.2 and −80 mV for K_V_1.4). The activation curves were fitted using the Boltzmann equation, as follows:

(1) $$G/G_{max}=1/\left(1+exp\left[\left(V_{1/2}-V\right)/\kappa \right]\right)$$

where *G* is the conductance, *V_1/2_* is the half maximum activation voltage, *V* is the testing voltage, and *κ* is the slope factor.

The steady-state inactivation of K_V_4.2 (Figure 5(d)) was studied using 1 s prepulse potentials ranging from −100 to 0 mV, followed by a 200 ms test pulse of +40 mV (Vh = −100 mV). The steady-state inactivation of K_V_1.4 (Figure 5(k)) was studied using 5 s prepulse potentials ranging from −80 to 0 mV, followed by a 1 s test pulse of +50 mV (Vh = −80 mV). The inactivation curves were fitted using the Boltzmann equation, as follows:

(2) $$G/G_{max}=R+\left(1-R\right)/\left(1+exp\left[\left(V-V_{1/2}\right)/\kappa \right]\right)$$

where *R* is the residual fraction of inactivation, *G* is the conductance, *V_1/2_* is the half maximum activation voltage, *V* is the testing voltage, and *κ* is the slope factor.

The falling phase of K_V_4.2 and K_V_1.4 currents were fitted with single (K_V_4.2) or two (K_V_1.4) exponential functions of the following form:

(3) $$I\left(t\right)=P+\sum \left[A_{n}\times exp\left(-t/\tau_{n}\right)\right]$$

where *P* is the residual persistent current, *A_n_* is amplitude of the n^th^ component, and *τ_n_* is time constant of the n^th^ component (shown in Figure 5(g,n,o)).

**Figure 1.**
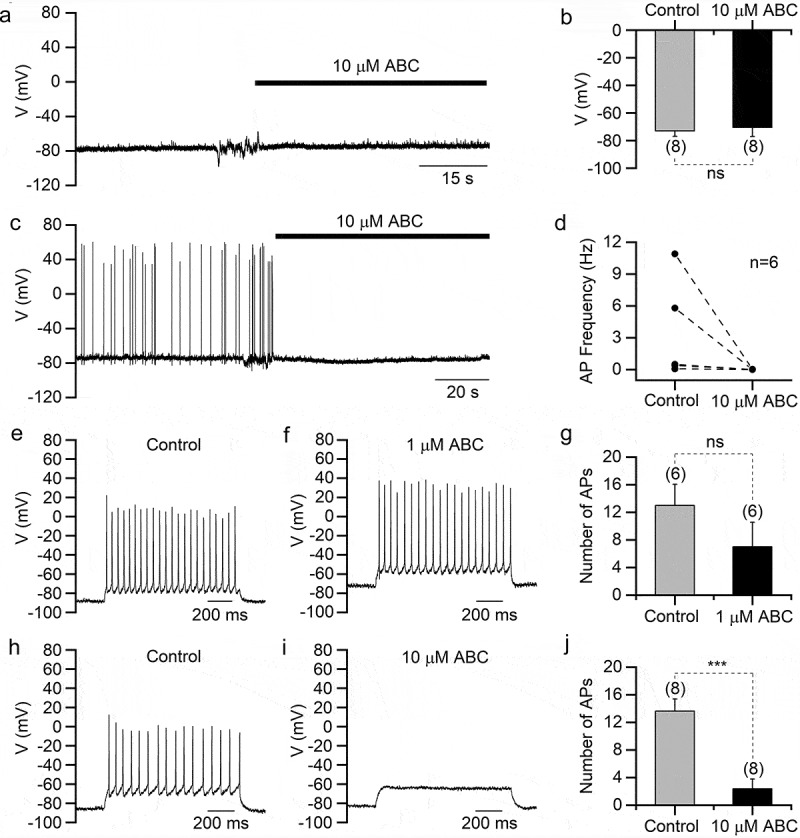
ABC294640 suppresses action potentials in hippocampal neurons. All recordings were performed in cultured mouse hippocampal neurons. (a) Representative current-clamp recording in a hippocampal neuron showing the resting membrane potential before and after ABC294640 application. (b) Averaged resting membrane potentials before and after ABC294640 application. (c) Representative current-clamp recording showing the spontaneous action potential before and after ABC294640 application. (d) Frequencies of spontaneous action potential before and after ABC294640 application. (e and f) Depolarizing current injection evoked action potentials in a hippocampal neuron before (e) and after (f) application of 1 μM ABC294640. (g) Numbers of evoked action potentials in (e) and (f). (h and i) Evoked action potentials recorded in a hippocampal neuron before (h) and after (i) application of 10 μM ABC294640. (j) Numbers of evoked action potentials in (h) and (i).

For current-clamp recordings, the bath solution contained 150 mM NaCl, 3.5 mM KCl, 1 mM MgCl_2_, 10 mM HEPES, 20 mM D-glucose, and 1.2 mM CaCl_2_ (pH 7.4 with NaOH), and the pipette solution contained 142 mM K-aspartate, 5 mM NaCl, 5 mM KCl, 2 mM MgCl_2_, 1 mM EGTA, 10 mM HEPES, and 4 mM Mg-ATP (pH 7.4 with KOH). To record resting membrane potential and spontaneous action potential (Figure 1(a–d)) I = 0 mode of current-clamp was used. To record the evoked action potential (Figure 1(e–j)), the cells were held with −15 pA current and injected with increasing current step pulses until a series of action potentials were induced. The liquid junction potentials were corrected online for the voltage-clamp recordings and corrected after experiments for the current-clamp recordings. The values of liquid junction potential (15.2 mV for recordings of sodium and potassium currents; 8.2 mV for recordings of calcium currents; 16.0 mV for current clamp recordings) were calculated using the Calculate Junction Potentials tool in the Clampex software (Molecular Devices) based on the composition of the bath and pipette solutions.

### Calcium imaging

SH-SY5Y cells and hippocampal neurons were used for calcium imaging. SH-SY5Y cells were incubated with 10 μM all-trans retinoic acid for 24 h before experiments. Cells were loaded with 1 μM Fura2-AM dye (Thermo Fisher Scientific) for 30 min at 37°C and washed twice using Tyrode’s solution. ABC294640 (30, 60, or 90 μM) and ionomycin (1 μM) were added at the indicated time. Calcium transients were captured by live-cell imaging using a calcium imaging system consisting of a DG-5 wavelength switcher (Sutter Instrument), an ORCA-Flash4.0 LT+ CMOS camera (Hamamatsu), and a Ti2 microscope (Nikon).

### Data analysis

All electrophysiological data were analyzed using Clampfit (Molecular Devices), Excel (Microsoft), or OriginPro (OriginLab). Numerical data are shown as mean ± SEM or mean + SEM. The t-test was used to calculate statistical significance. The dose-inhibition curves were fitted using the Hill equation, as follows:

(4) $$I/I_{0}=I/[1+{\left(X/IC50\right)}^{h}$$

where *X* is the concentration of ABC294640, and *h* is the Hill coefﬁcient. Calcium imaging data were analyzed using ImageJ and plotted using GraphPad.

## Results

### ABC294640 suppresses action potential firing in neurons

Because ABC294640 reportedly induces nervous system disorders, we first explored its effects on the electrophysiological characteristics of mouse hippocampal neurons. Using current-clamp recordings, we found that 10 µM ABC294640 did not significantly change the neuronal resting membrane potentials (Figure 1(a,b)). In spontaneously firing neurons, however, 10 µM ABC294640 almost eliminated spontaneous APs (Figure 1(c,d)). In neurons without spontaneous firing, we evoked APs using depolarizing current injection and tested the effect of ABC294640. We found that 1 µM ABC294640 slightly reduced the number of evoked APs, whereas 10 µM ABC294640 significantly suppressed firing (Figure 1(e–j)), which indicated that ABC294640 suppressed neuronal excitability.

### ABC294640 inhibits sodium and potassium currents in neurons

**Figure 2.**
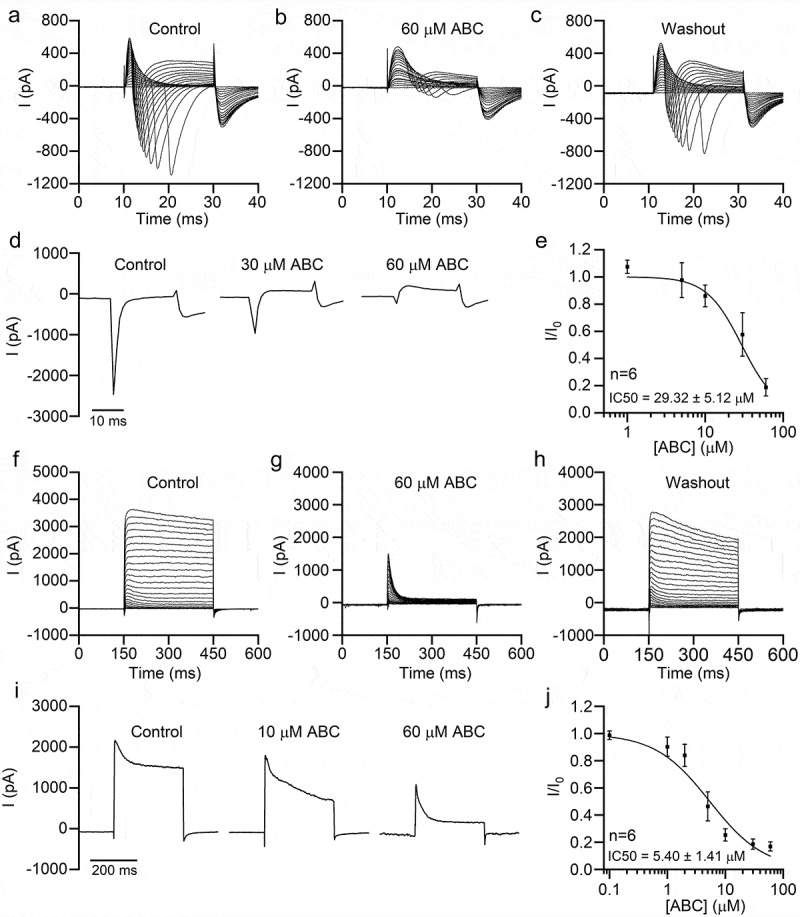
ABC294640 inhibits endogenous sodium and potassium currents in hippocampal neurons. Endogenous sodium (a–e) and potassium currents (f-j) were recorded in cultured mouse hippocampal neurons. (a–c) Representative sodium currents recorded before application (a), after application (b), and after washout (c) of 60 μM ABC294640. The currents were induced using 20 ms depolarizing steps (−80 to +20 mV, 5 mV step; Vh = −80 mV). (d) Representative sodium currents (elicited by depolarization to 0 mV, Vh = −80 mV) recorded with different concentrations of ABC294640 in bath solution. (e) Dose-inhibition curve of the effect of ABC294640 on endogenous sodium currents in hippocampal neurons. (f–h) Representative potassium currents recorded before application (f), after application (g), and after washout (h) of 60 μM ABC294640. The currents were induced using 300 ms depolarizing steps (−60 to +60 mV, 5 mV step; Vh = −80 mV). (i) Representative potassium currents (elicited by depolarization to +60 mV; Vh = −80 mV) recorded with different concentrations of ABC294640 in bath solution. (j) Dose-inhibition curve of the effect of ABC294640 on the persistent components of endogenous potassium currents (measured at the end of depolarization).

Voltage-gated sodium and potassium channels play fundamental roles in AP generation and neuronal excitability. Therefore, we next recorded Na^+^ and K^+^ currents in cultured mouse hippocampal neurons. As shown in Figure 2(a–e), ABC294640 dose-dependently inhibited neuronal Na^+^ currents with an IC50 value of 29.32 ± 5.12 μM, which was about half of the IC50 value of ABC294640 against SK2 [9]. Similarly, ABC294640 inhibited endogenous K^+^ currents in a dose-dependent manner (Figure 2(f–j)). Interestingly, we found that ABC294640 had a stronger inhibitory effect on the persistent components of K^+^ currents. In the presence of 60 μM ABC294640, the transient currents were largely retained, whereas the persistent currents were almost completely inhibited (Figure 2(g,i)). The IC50 of ABC294640 against the persistent K^+^ currents was 5.40 ± 1.41 μM, which indicated that ABC294640 had greater potency with K^+^ currents than with Na^+^ currents.

### ABC294640 inhibits sodium and potassium currents in SH-SY5Y cells

**Figure 3.**
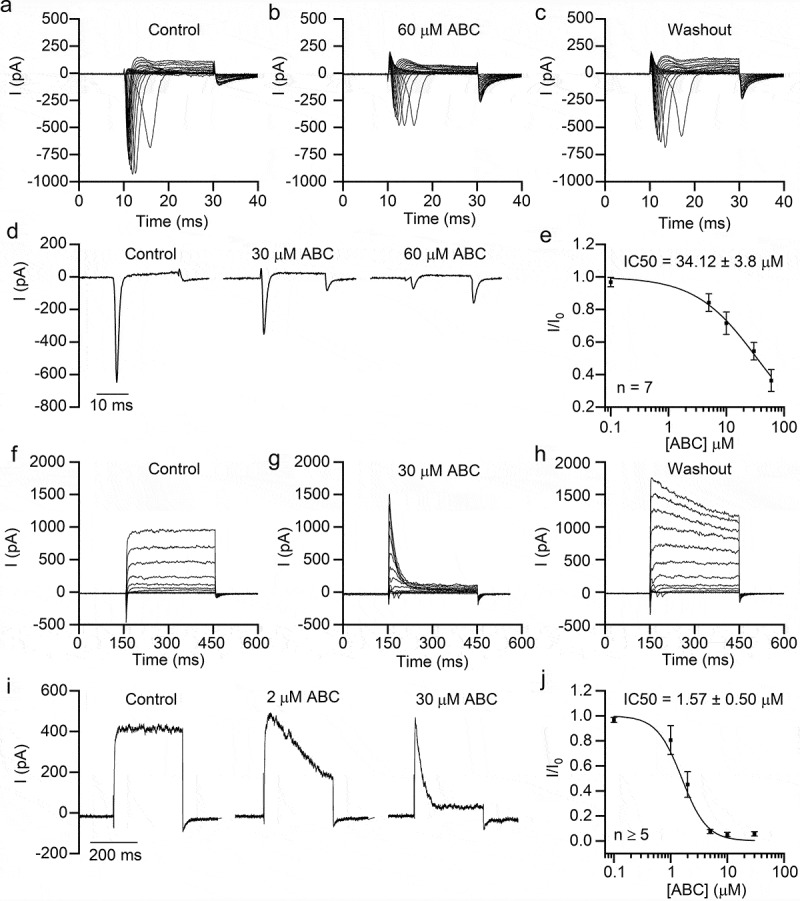
ABC294640 inhibits endogenous sodium and potassium currents in SH-SY5Y cells. Endogenous sodium currents (a–e) and potassium currents (f–j) were recorded in SH-SY5Y cells. (a–c) Representative sodium currents recorded before application (a), after application (b), and after washout (c) of 60 μM ABC294640. The currents were induced using 20 ms depolarizing steps between −80 and +20 mV (5 mV step; Vh = −80 mV). (d) Representative sodium currents (elicited by depolarization to −10 mV; Vh = −80 mV) recorded with different concentrations of ABC294640 in bath solution. (e) Dose-inhibition curve of the effect of ABC294640 on endogenous sodium currents in SH-SY5Y cells. (f–h) Representative potassium currents recorded before application (f), after application (g), and after washout (h) of 30 μM ABC294640. The currents were induced using 300 ms depolarizing steps between −60 and +80 mV (10 mV step; Vh = −80 mV). (i) Representative potassium currents (elicited by depolarization to +60 mV; Vh = −80 mV) recorded with different concentrations of ABC294640 in bath solution. (j) Dose-inhibition curve of the effect of ABC294640 on the persistent components of endogenous potassium currents (measured at the end of depolarization).

ABC294640 also inhibited endogenous Na^+^ currents in SH-SY5Y cells with an IC50 value of 34.12 ± 3.8 μM, which was close to that of hippocampal neurons (Figure 3(a–e)). Moreover, ABC294640 potently inhibited K^+^ currents in SH-SY5Y cells (Figure 3(f–j)). Similar to its effect on neuronal K^+^ currents, ABC294640 mainly inhibited the persistent component of SH-SY5Y K^+^ currents, with an IC50 value of 1.57 ± 0.50 μM (Figure 3(i–j)).

### ABC294640 inhibits calcium currents in SH-SY5Y cells

**Figure 4.**
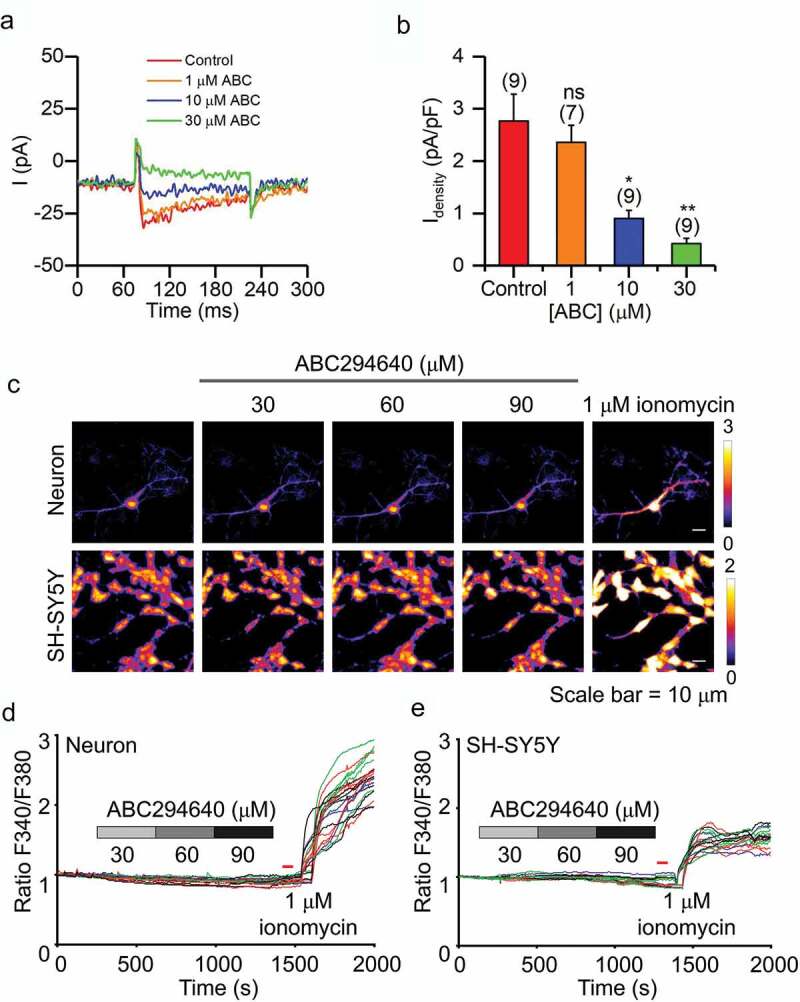
Effect of ABC294640 on calcium currents and intracellular calcium concentration. (a) Representative calcium currents recorded before and after application of 1, 10 or 30 μM ABC294640. Currents were recorded in SH-SY5Y cells and elicited by depolarization to +10 mV (Vh = −80 mV). (b) Calcium current density measured with different concentrations of ABC294640 in bath solution. (c) Representative calcium imaging with different concentrations of ABC292640 in bath solution in neurons and SH-SY5Y cells. (d and e) Time series of the fluorescence intensity ratio at excitation wavelengths of 340 and 380 nm during ABC294640 and ionomycin application in hippocampal neurons (d) and SH-SY5Y cells (e).

We also tested the effect of ABC294640 on calcium channels and intracellular calcium concentration. Voltage-gated Ca^2+^ currents in SH-SY5Y cells were notably decreased in the presence of 10 µM and 30 µM ABC294640 (Figure 4(a,b)). Surprisingly, in our calcium imaging experiments, we did not detect any effect of ABC294640 on intracellular calcium concentrations in either hippocampal neurons or SH-SY5Y cells (Figure 4(c–e)).

### ABC294640 has strong inhibitory effects on the voltage-gated potassium channels K_V_4.2 and K_V_1.4

**Figure 5.**
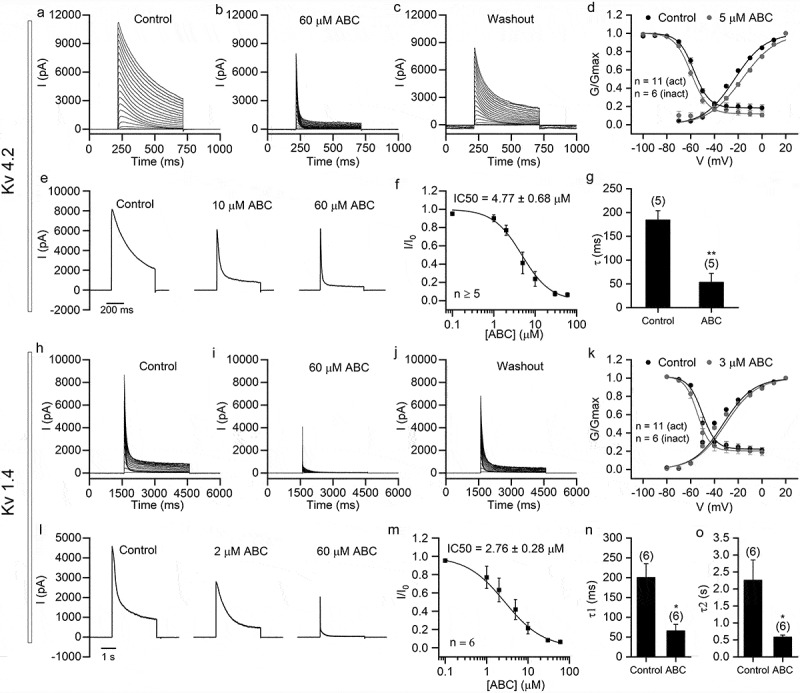
ABC294640 shows strong inhibition of K_V_4.2 and K_V_1.4 channels exogenously expressed in HEK293T cells. Recordings were performed in HEK293T cells transfected with *KCND2* (K_V_4.2; a–g) and *KCNA4* (K_V_1.4; h–o). (a–c) Representative K_V_4.2 currents recorded before application (a), after application (b), and after washout (c) of 60 μM ABC294640. The currents were elicited using 500 ms depolarizing voltage steps (−50 mV to +150 mV, 10 mV step; Vh = −80 mV). (d) The voltage dependence of activation and steady-state inactivation of K_V_4.2 before (black) and after application of 5 μM ABC294640 (gray). (e) Representative K_V_4.2 currents (elicited by depolarization to +100 mV; Vh = −80 mV) recorded with different concentrations of ABC294640 in bath solution. (f) Dose-inhibition curve of ABC294640 on persistent components of K_V_4.2 currents (measured at the end of depolarization). (g) Time constants of the falling phase of K_V_4.2 currents obtained by fitting with single exponential (see Methods). (h–j) Representative K_V_1.4 currents recorded before application (h), after application (i) and after washout (j) of 60 μM ABC294640. The currents were elicited by 3 s depolarizing voltage steps (−60 mV to +80 mV, 10 mV step; Vh = −80 mV). (k) The voltage dependence of activation and steady-state inactivation of K_V_1.4 before (black) and after application of 3 μM ABC294640 (gray). (l) Representative K_V_1.4 currents (elicited by depolarization to +60 mV; Vh = −80 mV) recorded with different concentrations of ABC294640 in bath solution. (m) Dose-inhibition curve of ABC294640 on persistent components of K_V_1.4 currents (measured at the end of depolarization). (n–o) Time constants of the fast component (τ1; n) and slow component (τ2; o) obtained by fitting the falling phase of K_V_1.4 currents with two-exponential function (see Methods).

Previous data have shown that, in both neurons and SH-SY5Y cells, ABC294640 potently inhibited K^+^ currents. Many types of potassium channels are expressed in these cells. To determine which particular potassium channels were inhibited, we next tested the effect of ABC294640 on HEK293T cells overexpressing specific types of potassium channels. We noticed that ABC292640 mainly inhibited persistent K^+^ currents in neurons and SH-SY5Y cells. For this reason, we first tested K_V_4.2 and K_V_1.4 – two A-type potassium channels with big transient currents and relative smaller persistent currents (Figure 5). ABC294640 strongly inhibited the persistent currents of both channels, with IC50 values of 4.77 ± 0.68 μM and 2.76 ± 0.28 μM, respectively (Figure 5(f,m)). The transient currents of both channels were less sensitive to ABC294640 – we still found large transient currents even in the presence of 60 μM ABC294640 (Figure 5(b,e,i,l)). A 5 μM concentration of ABC294640 right-shifted the activation curve and slightly left-shifted the steady-state inactivation curve of K_V_4.2. The V_1/2_ of activation was changed from −22.96 ± 0.73 mV to −16.71 ± 0.65 mV after ABC294640 application, whereas the V_1/2_ of inactivation was changed from −57.26 ± 1.04 mV to −59.42 ± 0.92 mV (Figure 5(d)). A 3 μM concentration of ABC294640 had little effect on the voltage dependence of K_V_1.4. The V_1/2_ of activation was changed from −32.50 ± 1.93 mV to −30.51 ± 1.68 mV after ABC294640 application, whereas the V_1/2_ of inactivation was changed from −50.37 ± 1.31 mV to −54.23 ± 0.63 mV (Figure 5(k)). These results suggested that ABC294640 had different influences on various voltage-gated channels. The time constants of the falling phase of both channels decreased significantly after ABC294640 application (Figure 5(g,n,o)), indicating that ABC294640 accelerated the inactivation of the two K_V_s.

### ABC294640 has a weaker inhibitory effect on BK and K2P2.1 channels

**Figure 6.**
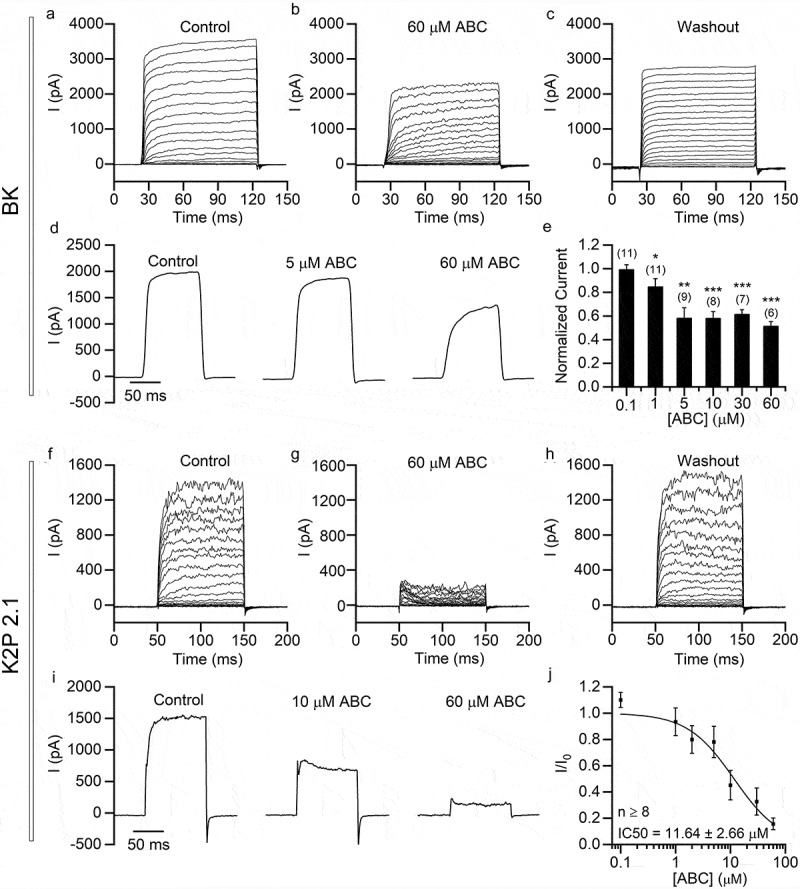
ABC294640 has relatively low inhibitory activity on BK and K2P2.1 channels exogenously expressed in HEK293T cells. Recordings were performed in HEK293T cells transfected with *KCNMA1* (BK; a–e) and *KCNK2* (K2P2.1; f-j). (a–c) Representative BK currents recorded before application (a), after application (b) and after washout (c) of 60 μM ABC294640. The currents were elicited using 100 ms depolarizing voltage steps (−50 mV to +150 mV, 10 mV step; Vh = −80 mV). (d) Representative BK currents (elicited by depolarization to +100 mV; Vh = −80 mV) recorded with different concentrations of ABC294640 in bath solution. (e) Current amplitudes (+100 mV) normalized to those measured in the absence of ABC294640. (f–h) Representative K2P2.1 currents recorded before application (f), after application (g) and after washout (h) of 60 μM ABC294640. The currents were elicited using 100 ms depolarizing voltage steps (−50 mV to +150 mV, 10 mV step; Vh = −80 mV). (i) Representative K2P2.1 currents (elicited by depolarization to +100 mV; Vh = −80 mV) recorded with different concentrations of ABC294640 in bath solution. (j) Dose-inhibition curve of the effect of ABC294640 on K2P2.1 currents.

Because ABC294640 mainly inhibits persistent K^+^ currents, we also tested two noninactivating K^+^ channels (i.e. BK and K2P2.1) in the HEK293T cells overexpressing each type of channel. ABC294640 weakly inhibited BK currents (Figure 6(a–e)). We detected less than 50% inhibition of the currents, even at an ABC294640 concentration of 60 µM (Figure 6(e)). The sensitivity of K2P2.1 currents was higher than that of BK currents, but it was lower than those of K_V_4.2 and K_V_1.4 (Figure 6(f–j)). The IC50 of ABC294640 on K2P2.1 was 11.64 ± 2.66 µM.

### The SK2 inhibitor K145 has a different effect on ion channels

**Figure 7.**
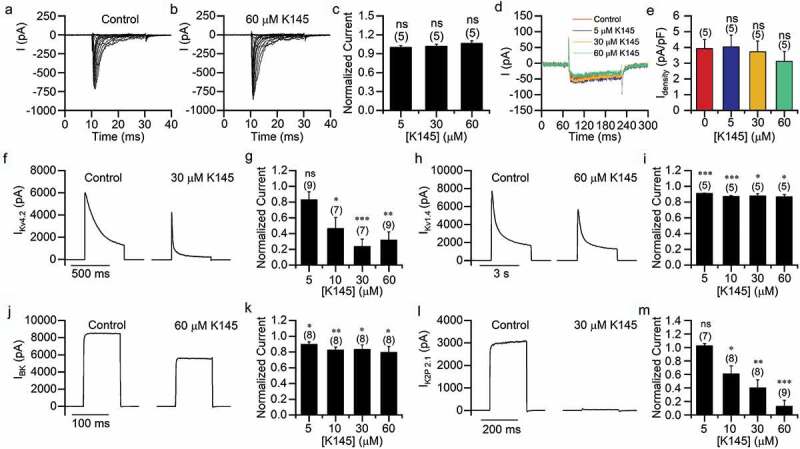
Effects of K145 on ion channels. (a–b) Representative sodium currents recorded in SH-SY5Y cells before (a) and after (b) application of 60 μM K145. The currents were induced using 20 ms depolarizing steps (−80 to +20 mV, 5 mV step; Vh = −80 mV). (c) Amplitudes of sodium current (elicited with 0 mV depolarization) normalized to those measured in the absence of K145. (d) Representative calcium currents recorded in SH-SY5Y cells before and after K145 application. (e) Calcium current densities in (D). (f–m) Potassium currents recorded in HEK293T cells expressing K_V_4.2 (f–g), K_V_1.4 (h–i), BK (j–k) and K2P2.1 (l–m). Representative currents (elicited using +100 mV depolarization) are shown in (f,h,j,l). Persistent currents normalized to those measured in the absence of K145 are shown in (g,i,k,m).

As an SK2 inhibitor, ABC294640 probably inhibits the ion channels by inhibiting SK2. To verify this hypothesis, we tested another selective and potent SK2 inhibitor K145 that had lower IC50 than ABC294640. Interestingly, K145 showed no inhibitory effect on endogenous Na^+^ and Ca^2+^ currents in SH-SY5Y cells (Figure 7(a–e)). In HEK293T cells overexpressing individual potassium channels, K145 dose-dependently inhibited K_V_4.2 and K2P2.1, but only slightly inhibited K_V_1.4 and BK channels (Figure 7(f–m)). The different inhibitory effect of K145 and ABC294640 probably indicated that ABC294640 inhibited ion channels in an SK2-independent manner.

### S1P does not alleviate the inhibitory effect of ABC294640 on potassium channels

**Figure 8.**
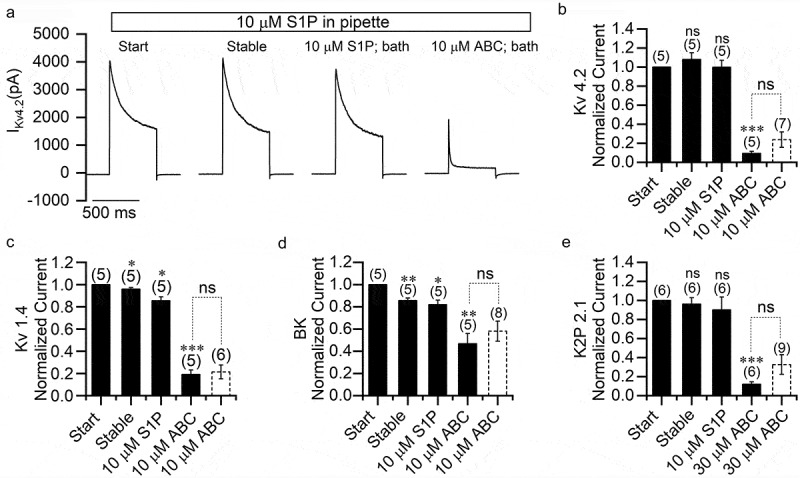
S1P does not eliminate the inhibitory effect of ABC294640 on potassium channels. (a) Representative K_V_4.2 currents recorded in transfected HEK293T cells with pipette solution containing 10 μM S1P. Current traces were obtained at the beginning of recording, after the current reached stability, after application of 10 μM S1P to the bath and after application of 10 μM ABC294640 to the bath. (b–e) Persistent currents of K_V_4.2 (b), K_V_1.4 (c), BK (d), and K2P2.1 (e) at different recording conditions normalized to the currents measured at the beginning of recordings. The white columns with the dotted frame were plotted using data adopted from Figs. 5 and 6 for comparison.

SK2 phosphorylates sphingosine to generate S1P. Thus, SK2 inhibition causes a reduction in S1P level. To further test the involvement of SK2 in the inhibition of ion channels by ABC294640, we added 10 μM S1P to the pipette and bath solution and recorded potassium currents in HEK293T cells overexpressing individual potassium channels (Figure 8). S1P had no effect on K_V_4.2 or K2P2.1 currents (Figure 8(a,b,e)), but it slightly inhibited K_V_1.4 and BK currents (Figure 8(c,d)). In the presence of S1P, ABC294640 still inhibited these potassium channel currents. The inhibitory effect on all four potassium channels was comparable to that without S1P.

### The inhibition of ion channels by ABC294640 is fast and reversible

**Figure 9.**
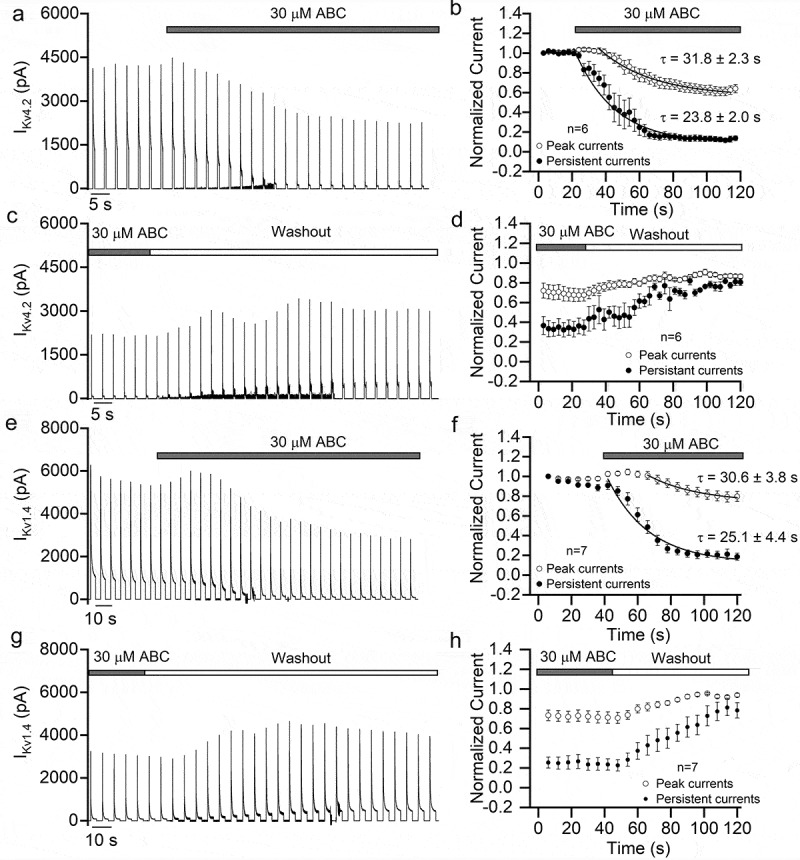
Time course of the inhibitory effect of ABC294640 on K_V_4.2 and K_V_1.4. (a) Continuous recording of K_V_4.2 currents before and during perfusion of 30 μM ABC294640. The currents were elicited using a series of +100 mV depolarization stimuli (500 ms duration, once every 3 s; Vh = −80 mV) and recorded in K_V_4.2-expressing HEK293T cells. (b) Time course of the changes in peak and persistent K_V_4.2 currents fitted with single exponential function. (c) Continuous recording of K_V_4.2 currents before and during washout of ABC294640. (d) Time course of the changes in K_V_4.2 currents before and during washout of ABC294640. (e) Continuous recording of K_V_1.4 currents before and during perfusion of 30 μM ABC294640. The currents were elicited using a series of +100 mV depolarization stimuli (3 s duration, once every 6 s; Vh = −80 mV) and recorded in K_V_1.4-expressing HEK293T cells. (f) Time course of the changes in peak and persistent K_V_1.4 currents fitted with single exponential function. (g) Continuous recording of K_V_1.4 currents before and during washout of ABC294640. (h) Time course of the changes in K_V_1.4 currents before and during washout of ABC294640.

The previous results suggested that the inhibition of ion channels by ABC294640 was not related to the SK2/S1P signaling pathway. Another possible mechanism of inhibition is direct action on the ion channels, so we tested the kinetics of this inhibition. In HEK293T cells overexpressing K_V_4.2 or K_V_1.4, we recorded K^+^ currents continuously and applied ABC294640 to the cells using a fast perfusion system. With this configuration, the extracellular concentration of ABC294640 reached the desired value in 5 s. As shown in Figure 9, ABC294640 began to inhibit the currents within a few seconds of the perfusion start, reaching its maximum inhibitory effect within 1 min. The time constants for inhibition of K_V_4.2 were 23.8 ± 2.0 s for persistent current and 31.8 ± 2.3 s for peak current (Figure 9(b)). In the case of K_V_1.4, the time constants for inhibition were 25.1 ± 4.4 s for persistent current, and 30.6 ± 3.8 s for peak current (Figure 9(f)). After ABC294640 washout, the current quickly recovered to about 80% of its original values in all cases (Figure 9(c,d,g,h)). The fast onset of inhibition and fast recovery suggested that ABC294640 acts directly on the channels.

## Discussion

As an SK2 inhibitor, ABC294640 showed interesting antitumor effects and therefore has attracted widespread attention. In one phase I clinical trial, ABC294640 was found to cause a series of neurological disorders. In the present study, we revealed the molecular mechanisms by which ABC294640 affected the nervous system. Specifically, it suppressed action potential firing in neurons and inhibited multiple ion channels. Action potential plays a fundamental role in signal transmission in the nervous system, and voltage-gated sodium and potassium channels are major contributors to action potential [14]. Therefore, the inhibition of sodium and potassium channels by ABC294640 likely has extensive effects on the nervous system.

In addition to sodium and potassium channels, we found that ABC294640 had an inhibitory effect on voltage-gated calcium channel (VGCC) in SH-SY5Y cells (Figure 4(a,b)). VGCC couple membrane depolarization to the influx of calcium, which in turn regulated neurotransmitter release, signal transduction, gene expression, and many other neuronal functions [15,16]. Therefore, ABC294640 may affect the functions of the nervous system by inhibiting VGCC activity. Interestingly, the intracellular Ca^2+^ concentrations were not affected by ABC294640, as shown in our calcium imaging in both neurons and SH-SY5Y cells (Figure 4(c–e)). This stability of intracellular Ca^2+^ concentration may occur in part because ABC294640 inhibited the AP. VGCC mainly opened during the AP, so ABC294640 may have confined neurons to a resting state and thus eliminated the calcium entry through VGCC. In addition, we found that, even in the presence of a very high ABC294640 concentration (90 μM), ionomycin significantly increased intracellular calcium signal. Ionomycin first acted on the intracellular calcium store [17,18], and thus our results suggested that ABC294640 did not inhibit ionomycin-induced calcium release from intracellular stores.

Calcium channels exerted crucial control over muscle contraction [19–21]. In skeletal muscle cells, VGCCs in the transverse tubule membranes directly acted on and activated ryanodine-sensitive Ca^2+^-release channels to initiate rapid excitation-contraction coupling [21,22]. In cardiac myocytes, Ca^2+^ influx through VGCCs activated Ca^2+^ release from the sarcoplasmic reticulum and triggered actomyosin activation and contraction [23,24]. Therefore, the inhibition of VGCCs by ABC294640 consequently may have affected muscle systems. Indeed, in the clinical trial, ABC294640 administration caused muscle spasm and muscle weakness in some patients [13], but it was not clear whether this abnormity in muscle function was due to calcium channel inhibition by ABC294640.

The inhibitory effects of ABC294640 on different ion channels varied widely, suggesting that the mechanisms of inhibition may differ. For the A-type potassium channels K_V_4.2 and K_V_1.4, ABC294640 mainly suppressed persistent currents. We tested the effect of ABC294640 on the voltage dependence of these two K_V_s. ABC294640 caused a marked right shift of the K_V_4.2 activation curve, but it had only a slight effect on the activation curve of K_V_1.4 and on the inactivation curves of both channels. Therefore, especially in the case of K_V_1.4, the inhibitory effect of ABC294640 did not function through the voltage dependence of the channels. ABC294640 significantly decreased the time constant of fast inactivation in both K_V_ types, indicating that the persistent current was inhibited largely because channel inactivation was accelerated. In the case of the noninactivating potassium channels BK and K2P2.1, ABC294640 inhibited the overall currents. This mechanism remains to be further studied in this case.

Many ion channels are expressed in non-neuronal cells, so the inhibition of ion channels by ABC294640 also may affect cells and tissues outside the nervous system. For example, BK channels expressed in smooth muscle cells play key roles in controlling vascular tone. They couple a local increase of intracellular Ca^2+^ with membrane hyperpolarization and vasodilation [25,26]. The inhibition of BK channels by ABC294640 may cause vascular constriction, which in turn may affect blood supply to the brain and induce clinical symptoms, such as dizziness.

Although the structurally distinct SK2 inhibitor K145 also strongly inhibited K_V_4.2 and K2P2.1 channels, its inhibitory effect on K_V_1.4 and BK was weak. For instance, 60 μM K145 inhibited the K_V_1.4 and BK currents only by about 20%, which was much less than 60 μM ABC294640. Notably, the inhibitory effect of K145 on SK2 was much stronger than that of ABC294640. More important, K145 had no effect on the endogenous sodium and calcium currents of SH-SY5Y cells. These results suggested that ABC294640 did not inhibit the channels through the SK2 pathway. In subsequent experiments, we found that S1P supplementation did not affect inhibition by ABC294640, further supporting this conclusion.

In addition, we found that ABC294640 inhibited ion channels quickly. The time course of inhibition was similar to that of the nonspecific potassium channel blockers 4-aminopyridine and TEA [27–29]. Therefore, we speculated that ABC294640 acted directly on the ion channel proteins. Moreover, we found that the inhibitory effect of ABC294640 on ion channels was reversible, as with many channel blockers. After washout, the currents were largely and quickly recovered. This reversibility was consistent with its effect on the nervous system. A clinical trial has shown that ABC294640-induced neuropsychiatric disorders resolved upon interruption or discontinuation of ABC294640 [13].

Although ABC294640 can cause nervous system disorders by affecting ion channels and AP, it also may have therapeutic effects in certain neurological diseases. Takasugi et al. discovered that ABC294640 could inhibit Aβ generation in N2a cells [30]. In another study, SK2 was found to localize to the nucleus of cultured cortical and striatal neurons to induce DNA double-strand breaks. In the brain of Huntington’s disease (HD) model BACHD mice, SK2 was upregulated and hyperphosphorylated. ABC294640 reduced DNA damage induced by ectopically expressed SK2 and exhibited neuroprotective effects in neuron models of HD [31].

Overall, the present study revealed the mechanism by which ABC2964640 interfered with nervous system function. These results have important implications for the further development of this compound. Structural modification to eliminate its effect on ion channels should reduce its side effects on the nervous system. Conversely, the development of specific ion channel inhibitors based on ABC294640 or its structural analogs will benefit neuroscience research and drug development for neurological diseases. New and more specific SK inhibitors should be identified in the future to inform the development of antitumor drugs.

## Abbreviations

SK1: Sphingosine kinase 1
SK2: Sphingosine kinase 2
S1P: Sphingosine-1-phosphate
APs: Action potentials
VGCCs: Voltage-gated calcium channels
